# Effect of home visiting support on maternal psychosocial needs and postnatal depression: emulating a target trial

**DOI:** 10.1136/bmjment-2026-302675

**Published:** 2026-06-19

**Authors:** Kaori Baba, Zui C Narita, Syudo Yamasaki, Tomohiro Shinozaki, Junko Niimura, Naomi Nakajima, Satoshi Yamaguchi, Gemma Knowles, Jordan Devylder, Satsuki Ayaya, Shin-ichiro Kumagaya, Daniel Stanyon, Nao Oikawa, Mariko Hosozawa, Miharu Nakanishi, Shuntaro Ando, Kiyoto Kasai, Mitsuhiro Miyashita, Mariko Hiraiwa-Hasegawa, Toshi A Furukawa, Vikram Patel, Susan M Sawyer, Atsushi Nishida

**Affiliations:** 1 Unit for Mental Health Promotion, Research Center for Social Science & Medicine, Tokyo Metropolitan Institute of Medical Science, Tokyo, Japan; 2 Graduate School of Nursing Science, St. Luke’s International University, Tokyo, Japan; 3 Department of Behavioral Medicine, National Center of Neurology and Psychiatry, Tokyo, Japan; 4 Interfaculty Initiative in Information Studies, The University of Tokyo, Tokyo, Japan; 5 Department of Biostatistics, School of Public Health, Graduate School of Medicine, The University of Tokyo, Tokyo, Japan; 6 Economic and Social Research Council (ESRC) Center for Society and Mental Health, Institute of Psychiatry, Psychology, and Neuroscience, King’s College London, London, UK; 7 Health Service and Population Research Department, Institute of Psychiatry, Psychology and Neuroscience, King’s College London, London, UK; 8 Silver School of Social Work, New York University, New York, New York, USA; 9 Tojisha-Kenkyu Department, Research Center for Advanced Science and Technology, The University of Tokyo, Tokyo, Japan; 10 Department of Pediatrics, Faculty of Medicine, Juntendo University, Tokyo, Japan; 11 Institute for Global Health Policy Research, Bureau of Global Health Cooperation, Japan Institute for Health Security, Tokyo, Japan; 12 Leiden University Medical Center, Leiden, The Netherlands; 13 Department of Psychiatric Nursing, Graduate School of Medicine, Tohoku University, Miyagi, Japan; 14 Department of Neuropsychiatry, Graduate School of Medicine, The University of Tokyo, Tokyo, Japan; 15 The International Research Center for Neurointelligence (WPI-IRCN), The University of Tokyo Institutes for Advanced Study (UTIAS), Tokyo, Japan; 16 School of Advanced Science, SOKENDAI (Graduate University for Advanced Studies), Kanagawa, Japan; 17 Department of Health Promotion and Human Behavior, Graduate School of Medicine / School of Public Health, Kyoto University, Kyoto, Japan; 18 Department of Global Health and Population at Harvard T.H. Chan School of Public Health, Harvard University, Boston, Massachusetts, USA; 19 Center for Adolescent Health, Royal Children's Hospital and Murdoch Children's Research Institute, Melbourne, Victoria, Australia; 20 Department of Pediatrics, University of Melbourne, Melbourne, Victoria, Australia

**Keywords:** Depressive Disorder, Mental Health Services

## Abstract

**Background:**

Adolescent and young adult (AYA) mothers often experience unmet psychosocial needs, and those under 25 years of age are at increased risk of perinatal depression. Although home visiting programmes may be beneficial, no controlled trial has evaluated a programme co-designed with first-time AYA mothers and grounded in their lived experiences.

**Objective:**

We evaluated the effect of the co-designed Early Partnership programme on perceived fulfilment of psychosocial needs and postnatal depressive symptoms among first-time AYA mothers.

**Methods:**

We emulated a target trial using data from a pragmatic historically controlled study in four Tokyo municipalities. Participants were primiparous women aged 16–25 years. The intervention group included 151 participants and the control group included 158 participants. The intervention group received intensive tailored home visits by multi-professional family support workers from pregnancy to 12 months postnatally, and the historical control group received publicly funded health and social services.

**Findings:**

Retention at 12 months was high in both groups (intervention group, 82.1%; control group, 82.9%). For the primary outcome analysis, the intervention group had greater improvement in perceived fulfilment of psychosocial needs at 6 months postnatally (difference in mean changes 4.16, 95% CI 1.35 to 6.97, Cohen’s *d* 0.40, 95% CI 0.13 to 0.67) and 12 months postnatally (difference in mean changes 3.93, 95% CI 0.63 to 7.23, Cohen’s *d* 0.39, 95% CI 0.06 to 0.72) than the control group. Postnatal depressive symptoms were lower in the intervention group at 6 months postnatally (mean difference −1.54, 95% CI −2.70 to −0.38, Cohen’s *d* −0.34, 95% CI −0.59 to −0.08) and 12 months postnatally (mean difference −1.55, 95% CI −2.74 to −0.36, Cohen’s *d* −0.34, 95% CI −0.61 to −0.07). Well-being also improved at each time point, but estimates were imprecise.

**Conclusions:**

The Early Partnership programme, co-designed with young mothers and delivered by multi-professional teams, was acceptable and effective in improving perceived fulfilment of psychosocial needs and postnatal depressive symptoms.

**Clinical implications:**

These findings suggest that a person-centred and relationship-based home visiting model, delivered through a non-stigmatising design, may represent a plausible model of maternal care.

**WHAT IS ALREADY KNOWN ON THIS TOPIC**

**WHAT THIS STUDY ADDS**

- Emulating a target trial, this study shows that the Early Partnership programme improved perceived fulfilment of psychosocial needs and reduced postnatal depressive symptoms among first-time mothers aged 16–25 years.

**HOW THIS STUDY MIGHT AFFECT RESEARCH, PRACTICE OR POLICY**

- A co-designed, non-judgmental, relationship-based home visiting model may represent a feasible approach to addressing psychosocial needs and postnatal depressive symptoms among young mothers.

## Background

 Adolescent and young adult (AYA) mothers continue to face substantial stigma,^1^ leading to social exclusion and limited access to appropriate healthcare. This contributes to persistent unmet psychosocial needs and reinforces health disparities,^1 2^ even in high-income countries where adolescent pregnancy is relatively rare. In addition to these challenges, AYA mothers are at increased risk of postnatal depression; this elevated risk is not confined to teenage mothers but extends to women under 25 years of age.^3^

Although perinatal services are widely available, in many settings routine perinatal care may be experienced as fragmented and risk-oriented, with limited emphasis on non-judgmental support.^4^ Such features of routine care have been identified as a potential barrier to consistent perinatal mental healthcare.^5^ Intensive home visiting programmes have been suggested as a potential approach to overcome such a barrier and are considered effective for certain outcomes.^6 7^ Established home visiting programmes, including Nurse–Family Partnership, have emphasised tailored support and trusting relationships between home visitors and mothers. Some studies have reported benefits of such programmes^8^,^10^; effects may be greater when support is delivered by a single home visitor rather than by a team,^8^ possibly reflecting the importance of continuity and a strong working alliance. In contrast, other studies have shown no clear benefit.^11 12^ This inconsistency may partly reflect changes in population risk or shifts in structural supports.^11^ These considerations highlight the importance of developing interventions that are responsive to the current needs and lived experiences of AYA mothers.

To address these limitations, the Early Partnership (EP) programme was co-designed with young mothers, municipal staff, researchers and community stakeholders to meet their unmet psychosocial needs and reduce the risk of postnatal depression. The EP programme offers intensive and multi-professional home visits by family support workers (FSWs), including public health nurses, social workers, psychologists and childcare professionals who provide non-judgmental, tailored support based on trusting relationships throughout the perinatal period. In the present study, we aimed to examine the effect of the EP programme on psychosocial needs and postnatal depression among first-time mothers aged 16–25 years, a group at elevated risk of postnatal depression. We emulated a target trial using data from a pragmatic, historically controlled trial across four municipalities in Tokyo, Japan. In target trial emulation, investigators explicitly specify the protocol of the hypothetical pragmatic randomised trial that would answer the causal question and then describe how each component of that protocol is mapped to the available data.^13^ This approach enabled us to translate our observational comparisons to well-defined effects that would be estimated from a randomised parallel-group trial.

## Materials and methods

### Study design

We emulated a target trial examining the effect of the EP programme, which incorporates intensive home visits into usual health and social services (table 1). As this study used a historically controlled design, participants were not randomised. All eligible primiparous women who submitted pregnancy notifications within the participating municipalities during each study period were systematically invited to participate in this service without knowledge of the inclusion of the EP programme, as this was considered a partial update of the whole service. Allocation concealment in the conventional trial sense does not apply because the personnel of the participating municipalities knew that there were added elements in the intervention period but selection bias is unlikely as all eligible women were invited. Participation rates were similar (60.3% (158/262) in the control period and 55.9% (151/270) in the intervention period), indicating no marked difference in enrolment mechanisms across calendar periods. The intervention and control groups corresponded to adjacent calendar years and publicly available statistics indicate relative stability in the underlying source population during these periods. Across Tokyo, the total number of pregnancy notifications was 103 440 in 2021, 98 745 in 2022 and 96 710 in 2023, indicating similar population sizes across periods.^14^ Age-specific fertility rates among women aged under 25 years were also comparable over these years, with rates among adolescents of 1.0, 0.9 and 0.9 per 1000 women, and among women in their early 20s of 6.3, 5.7 and 5.3 per 1000 women.^14^ The unemployment rate in Tokyo was likewise similar across the study period, at 3.0% in 2021, 2.6% in 2022 and 2.5% in 2023.^15^ The number of hospitals with obstetrics and gynaecology departments also remained essentially unchanged, from 86 in 2021 to 87 in 2022 and 87 in 2023.^16^ Although physician workforce statistics were available only every 2 years, the total number of physicians in Tokyo was broadly stable, with 48 578 physicians in 2020 and 48 072 in 2022.^17^ These data do not indicate major changes in the underlying source population or in selected contextual factors between periods. Taken together, these observations are consistent with broadly similar source populations across calendar periods. To address the possibility of residual differences in baseline characteristics between periods, our primary analysis adjusted for prespecified maternal characteristics which could act as potential confounders according to the literature.^18^,^21^ These procedures are described in the Statistical Analysis section.

**Table 1:** Specification and emulation of the target trial

Component	Target trial specification	Target trial emulation
Eligibility criteria	Primiparous women Aged 16–25 years Submitted pregnancy notifications Able to speak Japanese Planning to reside within the designated municipalities throughout the intervention period Provided written informed consent prior to enrolment Pregnancies not resulting in miscarriage, abortion, stillbirth or multiple pregnancies	Same as for the target trial, except for the differences in calendar year and month between the intervention group (August 2022–June 2023) and the control group (November 2021–July 2022)
Treatment strategies	Early Partnership (intervention): intensive, person-centred home visits tailored to the psychosocial needs of first-time mothers, beginning before 35 weeks’ gestation and continuing through 12 months postnatal Historical control: publicly funded health and social services	Same as for the target trial, except for the differences in calendar year and month between the intervention group (August 2022–June 2023) and the control group (November 2021–July 2022)
Treatment assignment	Participants are randomly assigned to a strategy at baseline and aware of the strategy to which they have been assigned	Treatment assignment was determined by calendar period rather than individual-level randomisation; to address the possibility of residual differences in baseline characteristics between periods, analyses adjusted for prespecified maternal characteristics identified from the literature as potential confounders
Outcomes	Primary outcome: Perceived fulfilment of psychosocial needs at T5 Secondary outcomes: Postnatal depressive symptoms at T3 to T5. Well-being at T2 to T5 Perceived fulfilment of psychosocial needs at T2 to T4	Same as for the target trial
Follow-up	Five time points: baseline (T1; <35 weeks’ gestation), 4 weeks after T1 (T2), 1 month postnatal (T3), 6 months postnatal (T4) and 12 months postnatal (T5)	Same as for the target trial, except for the differences in calendar year and month between the intervention group (August 2022–June 2023) and the control group (November 2021–July 2022)
Statistical analysis	Linear mixed models with a random intercept for individuals for perceived fulfilment of psychosocial needs, well-being and postnatal depressive symptoms. Multiple imputation by chained equations for missing data.	Same as for the target trial, except that the models adjusted for the aforementioned baseline covariates

The study is reported in accordance with the Transparent Reporting of Evaluations with Non-randomised Designs (TREND) statement checklist. All participants provided written informed consent.

### Eligibility criteria

Primiparous women aged 16–25 years who submitted pregnancy notifications from August 2022 to June 2023 for the EP group and from November 2021 to July 2022 for the control group were assessed for eligibility. Inclusion criteria were that participants spoke Japanese, planned to reside within the designated municipalities throughout the intervention period and provided written informed consent prior to enrolment. Exclusion criteria were pregnancies that resulted in miscarriage, abortion, stillbirth or multiple pregnancies.

### Treatment strategies

The EP programme was designed to identify the crucial psychosocial needs of first-time AYA mothers, foster collaborative partnerships between young mothers and professionals, and provide tailored and non-stigmatising support. Its key components included the following:

1. A person-centred approach to addressing the psychosocial needs of first-time AYA mothers, aiming to reduce the risk of future maternal depression.
2. Intensive home visits initiated during pregnancy (before 35 weeks’ gestation), with visit frequency tailored to participants’ needs (eg, once per week for those requiring a higher level of support).
3. A consistent and non-stigmatising assessment of maternal needs, conducted repeatedly throughout the programme.
4. Ongoing training and supervision for FSWs, within a multidisciplinary team comprising public health nurses, social workers, psychologists and childcare professionals. The training and supervision focused on ensuring that FSWs could respond comprehensively and in a timely manner to mothers’ psychosocial needs—including those related to time, finances, mental health and their physical health—while avoiding risk-oriented, stigmatising attitudes that are often found in conventional services.
5. Capability and consistency: Each participant was assigned a single FSW to ensure continuity of care and foster a trusting professional relationship. In this context, relationships were characterised by continuity with a designated FSW across pregnancy and the postnatal period, in contrast to the episodic and provider-changing contacts typical of usual care. While most support was provided by the assigned FSW, they could consult other professionals in the multidisciplinary team when necessary. A low caseload (up to 15 participants per worker) enabled timely and individualised support aligned with the programme’s core principles.

The historical control group received publicly funded health and social services available to all pregnant women on notification of pregnancy. These services included routine antenatal screening interviews, antenatal health education and infant health check-ups, as well as immunisations provided by community public health nurses. Participants in the EP group also continued to have access to all elements of usual municipal perinatal care, with the EP programme provided in addition to routine services.

### Participants and public involvement

Enhancing perinatal care and preventing postnatal depression by addressing mothers’ psychosocial needs through social support are recognised public health priorities. To avoid reproducing the risk-oriented and often stigmatising approaches common in conventional services, the co-design process of the EP programme involved two researchers (SA and SK) who were also individuals with lived experience. The final intervention design was developed in collaboration with the study’s Technical Advisory Group, which included experts in co-designed clinical research. To inform the design of the EP programme, we conducted a series of co-design activities between June 2021 and July 2022 with women who had experienced pregnancy at a young age and had found it difficult to seek help from existing services. These activities included workshops with approximately 10 women who had become pregnant at a young age and also had experience of substance use disorder and were receiving support at a recovery facility, conducted online and in person and facilitated by SA and SK. The discussions addressed two main questions: which aspects of existing services were experienced as difficult or alienating, and what kinds of expertise and training professionals would need in order to provide more acceptable support. These women described early risk-focused screening as shaming and stigmatising, which often discouraged further engagement with services. They also described a mismatch between the support they needed and the support typically provided. Many were seeking help with finances and practical support that were more overtly needed in the absence of family or other helpers, whereas services often focused on standard health guidance, such as vaccination schedules and infant feeding. These women indicated that this mismatch contributed to disengagement from services. These discussions directly informed the design of the EP programme. In particular, they led us to place greater emphasis on non-stigmatising initial contact, to incorporate lectures delivered by these women into staff training, and to add training for FSWs in practical financial planning and household cash-flow management. During the implementation of the EP programme, we organised meetings to share new skills and knowledge with community frontline workers and policymakers, with the goal of ensuring that the intervention was effectively integrated into existing maternal and child health services.

### Study outcomes

The primary outcome was perceived fulfilment of psychosocial needs at 12 months postnatally. Through the course of the co-design of the interventions, it became increasingly clear that there was no pre-existing scale to measure the unmet needs of AYA mothers and therefore we co-created a positively framed, non-stigmatising scale reflecting the mothers’ own priorities and experiences. The scale covered time, financial, physical health, mental health and life domains, with each item scored on a 10-point scale. The co-production with young mothers supports content validity. To further examine construct validity, we evaluated the scale in an external population-based cohort (Tokyo Teen Cohort; N=3171 parent–child pairs)^22^ and assessed its correlation with the General Health Questionnaire-28 (GHQ-28), observing a moderate inverse correlation (Pearson’s *r* −0.41, 95% CI −0.44 to −0.38). In addition, prior analyses have shown that lower scores on this scale are associated with increased odds of subsequent physical punishment of children.^23^ Together, these findings are consistent with the conceptualisation of the scale as reflecting unmet psychosocial needs associated with poorer mental health and adverse parenting practices. Furthermore, we pretested the scale and demonstrated its high internal consistency reliability (Cronbach’s α ranging from 0.85 to 0.90 across time points).^23^ Total scores ranged from 0 to 50, with higher scores indicating greater fulfilment of needs.

Secondary outcomes included postnatal depressive symptoms, well-being and fulfilment of psychosocial needs at earlier time points. Postnatal depressive symptoms were evaluated using the Edinburgh Postnatal Depression Scale (EPDS).^24^ Internal consistency was high, with Cronbach’s α ranging from 0.84 to 0.87 across time points. To contextualise the findings in our sample against general postpartum populations, we also calculated the prevalence of probable postnatal depression at 1 month postnatally using the established Japanese EPDS cut-off score ≥9.^24^ Well-being was measured using the WHO-5 Well-Being Index.^25^ Internal consistency was high, with Cronbach’s α ranging from 0.88 to 0.90 across time points.

### Follow-up

Self-report questionnaires assessed perceived fulfilment of psychosocial needs and well-being at five time points: baseline (T1; < 35 weeks’ gestation), 4 weeks after T1 (T2), 1 month postnatal (T3), 6 months postnatal (T4) and 12 months postnatal (T5). Postnatal depressive symptoms were assessed at the postnatal stages (T3–T5). In the EP group, participants’ responses were shared with FSWs, with their consent, to develop tailored support plans. Participants could voluntarily withdraw at any time. FSWs systematically documented interactions, including frequency and mode of engagement to monitor service utilisation and ensure continuity of care.

### Sample size

The sample size of 150 participants per group was determined using G*Power V.3.1.9.2, targeting 95% power with a two-sided α of 0.05 under the expected effect size set at 0.30 for perceived fulfilment of psychosocial needs based on prior research.^26^ As these numbers assumed random allocation between the groups (ie, as if the target trial were actually conducted), the actual power following confounding adjustment would have been decreased by the covariate imbalance between the groups. Nevertheless, we adopted this approach for the feasibility of the study and for conservative sizing with low nominal *β*.

### Statistical analysis

All analyses were conducted using R V.4.5.1. Baseline characteristics of the intervention and control groups were summarised for each group, and between-group differences were described using p values and standardised mean differences (SMDs) with 95% CIs. The 95% CIs for SMDs were estimated using percentile bootstrap methods with 1000 resamples. In addition, to assess whether the composition of participants retained at each assessment point differed between groups over time, we summarised baseline characteristics separately for participants retained at each assessment point. Primary analyses used repeated-measures models incorporating all follow-up time points for each participant.

For perceived fulfilment of psychosocial needs and well-being, we used linear mixed models for change from baseline scores to examine group differences in outcome trajectories over time by including group, time (dummy variables for T2–T5) and their interaction term as fixed effects. A random intercept was included to account for individual variability. To address the possibility of residual differences in baseline characteristics between periods, we adjusted for age, gestational week at pregnancy notification, educational attainment,^21^ employment status,^21^ income,^21^ marital status,^20^ living arrangement,^18^ return to the maternal home for childbirth,^19^ social support^20^ and the baseline value of each outcome. For postnatal depressive symptoms, we examined between-group differences during the postnatal period (T3–T5) using linear mixed models, adjusting for the aforementioned covariates and well-being, which was assumed to serve as a proxy for antenatal depressive symptoms at baseline. Because EPDS assesses postnatal depressive symptoms and was therefore administered only at the postnatal stages (T3–T5), we estimated between-group differences at each postnatal time point rather than change from baseline.

Dropout was defined as discontinuation of participation resulting from relocation during the study period. Participants were not considered dropouts if they missed a follow-up survey but continued to receive the intervention and were subsequently sent a questionnaire. In line with these definitions, all individuals who did not meet the criteria for dropout were treated as retained.

All participants were included in the analyses. We handled missing data via multiple imputation by chained equations using random forest algorithms. All relevant variables were included in the imputation model, and 20 imputed datasets were generated. Each dataset was analysed separately, and the results were combined according to Rubin’s rules. The prevalence of probable postnatal depression at 1 month postnatally was also estimated using the same multiply imputed datasets. As a sensitivity analysis for missing outcome data, we repeated the main analyses without multiple imputation, using the same linear mixed models fitted under full-information maximum likelihood (FIML) with all available observed data. Standardised effect sizes in the primary analysis were calculated as Cohen’s *d* by dividing the adjusted between-group mean difference at each time point by the pooled SD of the outcome at that time point. The pooled SD was computed within each imputed dataset, and standardised effect sizes were combined across the 20 multiply imputed datasets using Rubin’s rules.

## Results

**Figure 1:**
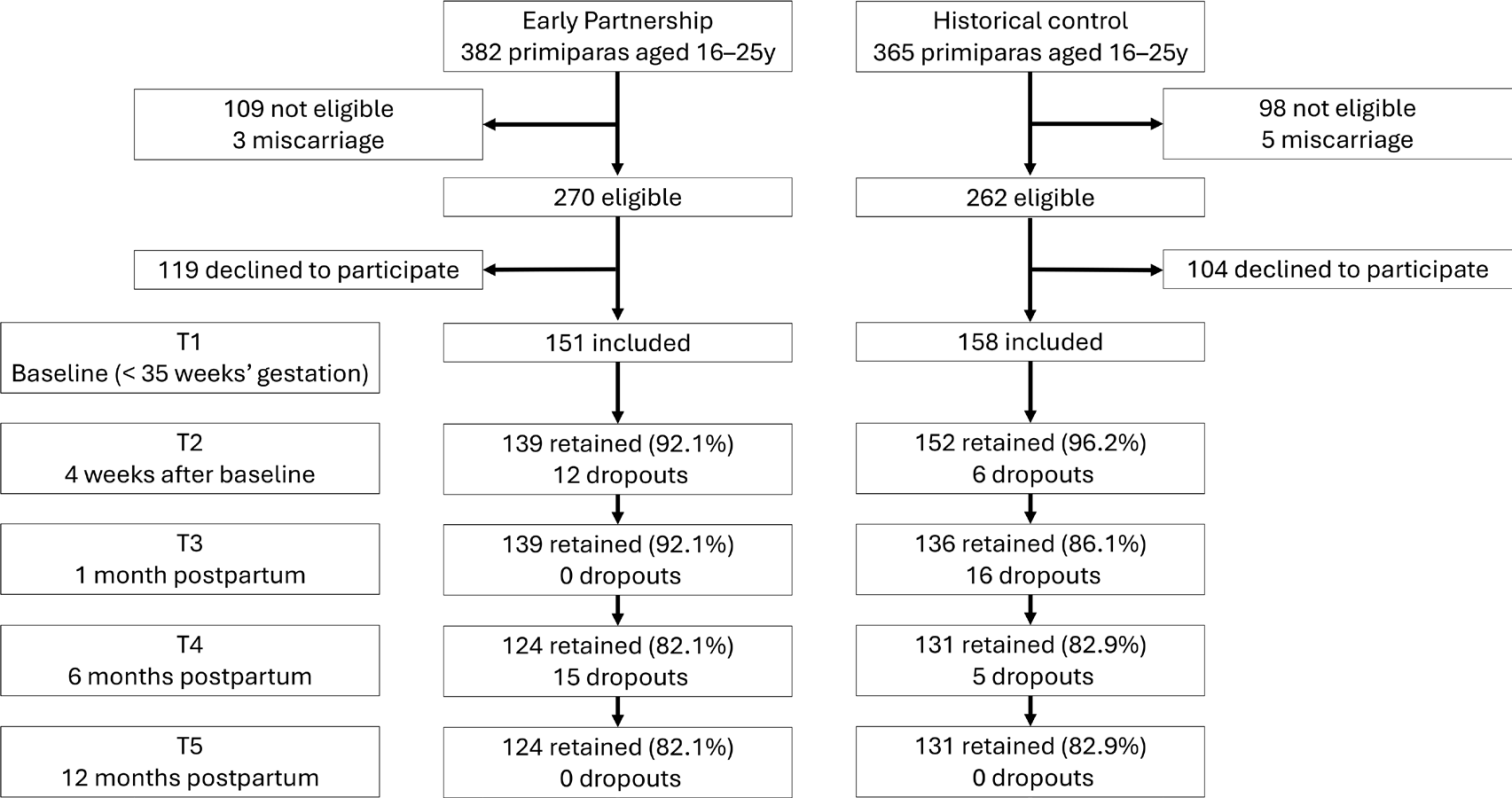
Study flow diagram.

A total of 309 participants were included: 151 in the EP group and 158 in the historical control group (figure 1). In the EP group, FSWs delivered a mean of 54.3 contacts per participant over the intervention window (mean 14.9 months from enrolment to 12 months postnatal). On average, this included 8.5 face-to-face consultations (home or centre-based), 17.2 telephone contacts, 29.3 online contacts and 2.2 other contacts (eg, letters). Table 2 shows the baseline characteristics of participants. The mean (SD) age was 23.5 (2.0) years in the EP group and 23.2 (1.8) years in the historical control group; other baseline variables were also comparable between groups. Baseline characteristics of participants retained at each assessment point are presented in Online supplemental file 1; these characteristics were broadly similar between groups across time points. In the EP group, retention rates were 92.1% at T2, 92.1% at T3, 82.1% at T4 and 82.1% at T5. In the historical control group, the corresponding rates were 96.2%, 86.1%, 82.9% and 82.9%, respectively. At 1 month postnatally, the prevalence of probable postnatal depression was 17.3% in the EP group and 21.0% in the historical control group.

**Table 2:** Summary of baseline characteristics

	Early partnership (n=151)	Historical control (n=158)	P value	SMD (95% CI)
Age, mean (SD)	23.5 (2.01)	23.2 (1.78)	0.14	0.17 (0.01 to 0.41)
Gestational week at pregnancy notification, mean (SD)	14.8 (8.03)	13.4 (7.10)	0.11	0.18 (0.01 to 0.41)
Educational attainment, n (%)			0.10	0.25 (0.07 to 0.49)
High school or lower	47 (31.1)	41 (25.9)		
Junior or technical college	49 (32.5)	70 (44.3)		
Bachelor’s degree or higher	55 (36.4)	47 (29.7)		
Employment status, n (%)			0.44	0.10 (0.00 to 0.32)
Unemployed or working ≤3 days/week	51 (33.8)	61 (38.6)		
Working ≥4 days/week	100 (66.2)	97 (61.4)		
Income, JPY, n (%)			0.50	0.18 (0.08 to 0.42)
<4 000 000	47 (31.1)	54 (34.2)		
4 000 00 0–6 999 999	48 (31.8)	58 (36.7)		
≥7 000 000	50 (33.1)	42 (26.6)		
Missing	6 (4.0)	4 (2.5)		
Marital status, n (%)			0.28	0.14 (0.01 to 0.37)
Married	109 (72.2)	104 (65.8)		
Not married	42 (27.8)	54 (34.2)		
Living arrangements, n (%)			0.91	0.04 (0.00 to 0.28)
Living with someone	141 (93.4)	146 (92.4)		
Living alone	10 (6.6)	12 (7.6)		
Returned to the maternal home for childbirth, n (%)			0.55	0.13 (0.01 to 0.33)
No	93 (61.6)	101 (63.9)		
Yes	58 (38.4)	56 (35.4)		
Missing	0 (0)	1 (0.6)		
Social support, mean (SD)	4.34 (2.37)	3.99 (2.25)	0.19	0.15 (0.01 to 0.38)
Missing	2 (1.3)	1 (0.6)		
Perceived fulfilment of psychosocial needs, mean (SD)	28.3 (8.92)	27.5 (10.6)	0.44	0.09 (0.01 to 0.31)
Well-being, mean (SD)	60.2 (21.7)	62.0 (21.2)	0.48	0.08 (0.00 to 0.32)

JPY, Japanese yen.SMD, standardised mean difference.

**Figure 2:**
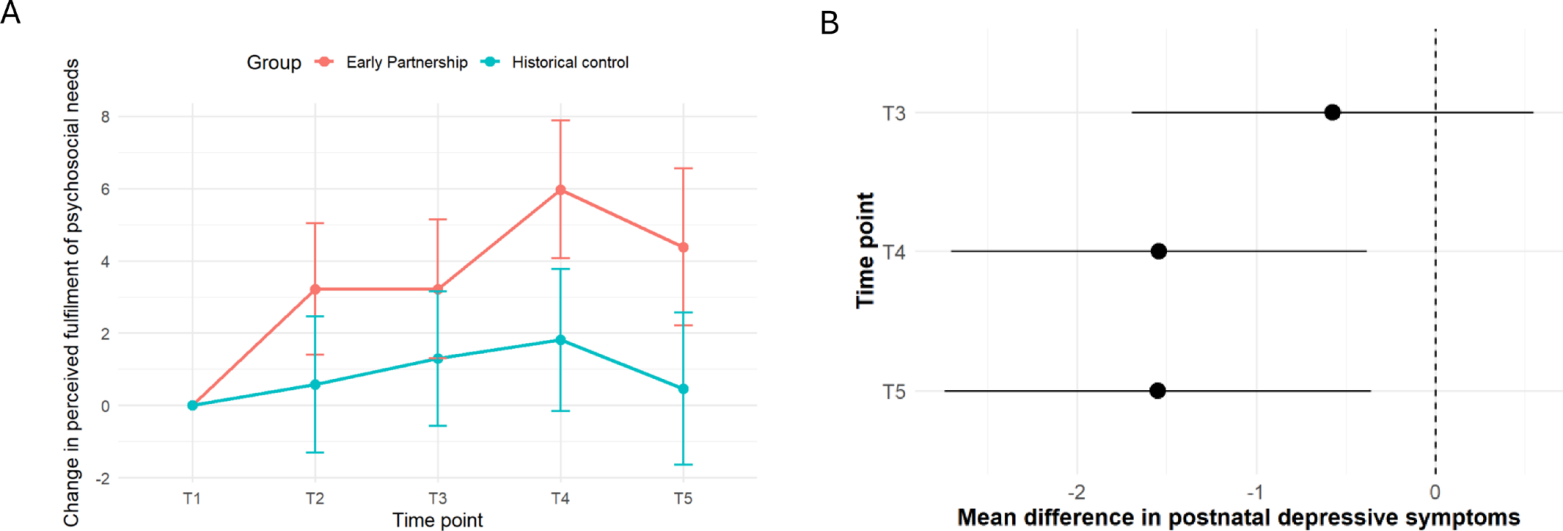
(A) Changes in perceived fulfilment of psychosocial needs. (B) Between-group difference in postnatal depressive symptoms (values <0 indicate lower symptom levels in the Early Partnership group). T1, <35 weeks’ gestation; T2, 4 weeks after T1; T3, 1 month postnatal; T4, 6 months postnatal; T5, 12 months postnatal.

For the primary outcome analysis, the EP group showed a greater improvement in perceived fulfilment of psychosocial needs than the historical control group at T5 (difference in mean changes 3.93, 95% CI 0.63 to 7.23, Cohen’s *d* 0.39, 95% CI 0.06 to 0.72) (figure 2A and supplementary table 4). A similar between-group effect was already evident at T4 (difference in mean changes 4.16, 95% CI 1.35 to 6.97, Cohen’s *d* 0.40, 95% CI 0.13 to 0.67), but without strong evidence at T3. As shown in figure 2B and supplementary table 5, postnatal depressive symptoms were lower in the EP group compared with the historical control group at T5 (mean difference −1.55, 95% CI −2.74 to −0.36, Cohen’s *d* −0.34, 95% CI −0.61 to −0.07). This difference emerged at T4 (mean difference −1.54, 95% CI −2.70 to −0.38, Cohen’s *d* −0.34, 95% CI −0.59 to −0.08), without strong evidence at T3 (figure 2B and supplementary table 5). Well-being improved over time in the EP group compared with the historical control group, but the CIs were wide relative to the effect size, resulting in insufficient precision to support a definite interpretation (supplementary table 6).

Sensitivity analyses using the same linear mixed models fitted under FIML without multiple imputation showed similar results to the primary analyses (supplementary figure 1 and supplementary tables 7–9).

## Discussion

Using a target trial emulation applied to data from a pragmatic, historically controlled trial, we translated our observational comparisons into well-defined effects that could be estimated in a randomised parallel-group trial. We clarified the differences between the specified trial and the emulated trial, thereby illustrating the potential sources of bias (table 1). We then applied appropriate adjustments to address these biases, thus clarifying the estimand. Our analysis provides evidence supporting the effect of the EP programme in enhancing perceived fulfilment of psychosocial needs and yielding lower levels of postnatal depressive symptoms among first-time AYA mothers aged 16–25 years, a population at elevated risk of postnatal depression. In contrast to features of routine perinatal care that may be experienced as fragmented or judgmental, the EP programme provided continuous, multi-professional home visits through a non-stigmatising and person-centred approach tailored to the diverse unmet needs of AYA mothers.^4^ Co-designed with AYA mothers, municipal staff and stakeholders, and delivered by a multidisciplinary team, the programme set out to foster relationships between mothers and FSWs from pregnancy through the postnatal period.

Pregnancy among young women remains highly stigmatised, and many mothers avoid formal services due to fear of judgement. Within this context, the person-centred and non-stigmatising design of the EP programme may help explain its acceptability, as reflected in sustained engagement. The finding that perceived fulfilment of psychosocial needs improved more among participants in the EP group may be interpreted as evidence that the programme addressed gaps within standard perinatal services. Such approaches are intended to enable mothers to engage more openly with professionals and to receive timely responses to psychosocial challenges that may otherwise remain unrecognised. In this sense, perceived fulfilment of psychosocial needs provides a meaningful indicator of the responsiveness and adequacy of perinatal support. The present study further found lower levels of postnatal depressive symptoms among participants in the EP group, an outcome of particular importance given the elevated risk of postnatal depression among mothers under 25 years of age.^3^ Overall, our findings highlight the importance of non-judgmental and relationship-based models of care in improving both psychosocial support and maternal mental health among AYA mothers.

Beyond statistical significance, the clinical relevance of the observed effect sizes warrants consideration. The EP programme was associated with small-to-moderate and consistent improvements in perceived fulfilment of psychosocial needs (Cohen’s *d* 0.40 at T4 and 0.39 at T5), representing a meaningful shift in how young mothers experience the adequacy and responsiveness of perinatal support. The observed reduction in postnatal depressive symptoms was small in magnitude and consistent across postnatal assessments (Cohen’s *d* −0.34 at both T4 and T5), which is larger than the pooled effect sizes reported for universal psychological preventive interventions in a recent meta-analysis.^27^ In this study population of mothers under 25 years of age, the prevalence of probable postnatal depression at 1 month postnatally was higher in both groups than that reported during the COVID-19 pandemic in a metropolitan area of Japan (15.8%), using exactly the same cut-off approach.^28^ This comparison supports the relevance of focusing on mothers under 25 years of age as a population at increased risk of postnatal depression. Although between-group differences in well-being did not reach conventional thresholds for statistical significance, consistently higher scores in the EP group across postnatal time points are reassuring and suggest that improvements in psychosocial needs may translate into broader gains in subjective well-being over time.

This study has limitations. First, enrolment among eligible AYA mothers was 58.1% overall, indicating substantial non-participation at entry and potential selection bias. Greater attention to enhancing recruitment remains a priority for home visiting studies, which may reflect the stigma of early pregnancy. For example, a study in England reported enrolment rates of 11% to 68% among eligible adolescent mothers, with lower uptake in areas with high adolescent pregnancy rates.^29^ In the present study, approximately 20% of participants dropped out during the follow-up. While the attrition was lower than that reported in similar research of adolescent parents,^30^ loss to follow-up may have introduced selection bias. As the EP programme was geographically limited to four Tokyo municipalities, expanding programme coverage would likely reduce dropout due to relocations. Second, our results may be influenced by unmeasured confounding. Although we assumed conditional exchangeability between groups by adjusting for potential confounders, residual confounding cannot be ruled out. Third, the start of follow-up, eligibility and treatment allocation, that is, ‘time zero’, was aligned in each participant; however, there were differences in calendar year and month between the intervention group (August 2022–June 2023) and the control group (November 2021–July 2022). These calendar time differences may have introduced temporal bias, particularly given that the study period overlapped with the COVID-19 pandemic. Fourth, the outcomes were based on self-reported questionnaires and may therefore be influenced by participants’ subjective appraisal of their experiences. Collectively, these limitations may offer alternative explanations for the observed effects and, given the non-experimental design, suggest that the findings should be interpreted cautiously. Fifth, the follow-up was limited to 12 months postnatally. Longer-term evaluation is needed to examine the durability of outcomes.

## Conclusion

In conclusion, the co-designed EP programme delivered by a multidisciplinary team was feasible and effective in improving perceived fulfilment of psychosocial needs and reducing postnatal depressive symptoms among AYA mothers. These findings support the value of routine, relationship-based, non-judgmental care as an alternative to screening-based approaches for this population.

## Supplementary material

10.1136/bmjment-2026-302675 online supplemental file 1

## Data Availability

Data are available upon reasonable request.
