# Supplementary material for: Effect of home visiting support on maternal psychosocial needs and postnatal depression: emulating a target trial
Source: BMJ Ment Health. 2026 Jun 19;29(1):e302675. doi: 10.1136/bmjment-2026-302675 (PMC13289090; doi:10.1136/bmjment-2026-302675)
Supplement: online supplemental file 1 [file bmjment-29-1-s001.pdf]

Supplementary Figure 1 Sensitivity analysis with missing data handled using full information maximum likelihood instead of multiple imputation: (A) Changes in perceived fulfillment of psychosocial needs; (B) Between-group difference in postnatal depressive symptoms (values < 0 indicate lower symptom levels in the Early Partnership group). T1, < 35 weeks' gestation; T2, 4 weeks after T1; T3, 1 month postnatal; T4, 6 months postnatal; T5, 12 months postnatal.

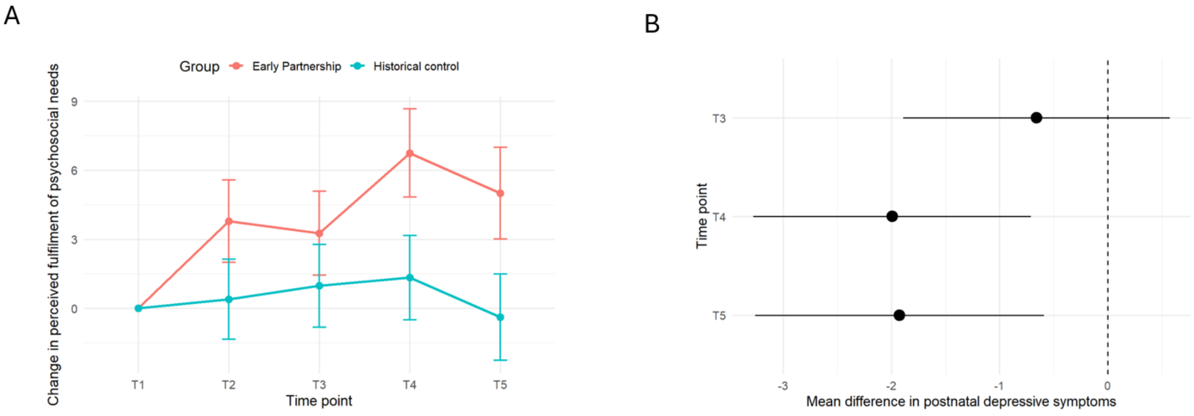

Supplementary Table 1 Summary of baseline characteristics of the participants included at T2.

	Early Partnership (*n* =139)	Historical control (*n* = 152)	P-value	SMD [95% CI]
Age, mean (SD)	23.5 (2.06)	23.2 (1.79)	0.21	0.15 [0.01–0.41]
Gestational week at pregnancy notification, mean (SD)	15.0 (8.22)	13.5 (7.20)	0.09	0.20 [0.01–0.43]
Educational attainment, n (%)			0.12	0.24 [0.09–0.49]
High school or lower	42 (30.2)	39 (25.7)		
Junior or technical college	45 (32.4)	67 (44.1)		
Bachelor's degree or higher	52 (37.4)	46 (30.3)		
Employment status, n (%)			0.37	0.12 [0.01–0.37]
Unemployed or working ≤ 3 days/week	46 (33.1)	59 (38.8)		
Working ≥ 4 days/week	93 (66.9)	93 (61.2)		
Income, JPY, n (%)			0.54	0.17 [0.08–0.48]
< 4,000,000	43 (30.9)	50 (32.9)		
4,000,000–6,999,999	44 (31.7)	57 (37.5)		
≥ 7,000,000	47 (33.8)	41 (27.0)		
Missing	5 (3.6)	4 (2.6)		
Marital status, n (%)			0.37	0.12 [0.01–0.36]
Married	101 (72.7)	102 (67.1)		
Not married	38 (27.3)	50 (32.9)		
Living arrangements, n (%)			0.79	0.06 [0.00–0.31]
Living with someone	131 (94.2)	141 (92.8)		
Living alone	8 (5.8)	11 (7.2)		
Returned to the maternal home for childbirth, n (%)			0.62	0.12 [0.01–0.31]
No	87 (62.6)	96 (63.2)		
Yes	52 (37.4)	55 (36.2)		
Missing	0 (0.0)	1 (0.7)		
Social support, mean (SD)	4.35 (2.37)	4.08 (2.23)	0.32	0.12 [0.01–0.34]
Missing	2 (1.4)	1 (0.7)		
Perceived fulfillment of psychosocial needs, mean (SD)	28.6 (9.06)	27.7 (10.5)	0.44	0.09 [0.00–0.31]
Well-being, mean (SD)	60.3 (21.5)	62.3 (21.2)	0.45	0.09 [0.00–0.32]

SD, standard deviation; JPY, Japanese yen.

Supplementary Table 2 Summary of baseline characteristics of the participants included at T3.

	Early Partnership (*n* =139)	Historical control (*n* = 136)	P-value	SMD [95% CI]
Age, mean (SD)	23.5 (2.06)	23.2 (1.83)	0.18	0.16 [0.01–0.41]
Gestational week at pregnancy notification, mean (SD)	15.0 (8.22)	13.7 (7.40)	0.15	0.17 [0.01–0.43]
Educational attainment, n (%)			0.17	0.23 [0.06–0.50]
High school or lower	42 (30.2)	33 (24.3)		
Junior or technical college	45 (32.4)	59 (43.4)		
Bachelor’s degree or higher	52 (37.4)	44 (32.4)		
Employment status, n (%)			0.37	0.12 [0.01–0.36]
Unemployed or working ≤ 3 days/week	46 (33.1)	53 (39.0)		
Working ≥ 4 days/week	93 (66.9)	83 (61.0)		
Income, JPY, n (%)			0.60	0.16 [0.09–0.45]
< 4,000,000	43 (30.9)	45 (33.1)		
4,000,000–6,999,999	44 (31.7)	50 (36.8)		
≥ 7,000,000	47 (33.8)	38 (27.9)		
Missing	5 (3.6)	3 (2.2)		
Marital status, n (%)			0.44	0.11 [0.01–0.34]
Married	101 (72.7)	92 (67.6)		
Not married	38 (27.3)	44 (32.4)		
Living arrangements, n (%)			1.00	0.01 [0.00–0.28]
Living with someone	131 (94.2)	128 (94.1)		
Living alone	8 (5.8)	8 (5.9)		
Returned to the maternal home for childbirth, n (%)			0.54	0.13 [0.01–0.33]
No	87 (62.6)	88 (64.7)		
Yes	52 (37.4)	47 (34.6)		
Missing	0 (0.0)	1 (0.7)		
Social support, mean (SD)	4.35 (2.37)	4.14 (2.27)	0.45	0.09 [0.00–0.31]
Missing	2 (1.4)	1 (0.7)		
Perceived fulfillment of psychosocial needs, mean (SD)	28.6 (9.06)	27.5 (10.7)	0.36	0.11 [0.01–0.34]
Well-being, mean (SD)	60.4 (21.5)	62.2 (21.4)	0.48	0.09 [0.01–0.33]

SD, standard deviation; JPY, Japanese yen.

Supplementary Table 3 Summary of baseline characteristics of the participants included at T4 and T5 (exactly same samples).

	Early Partnership (*n* =124)	Historical control (*n* = 131)	P-value	SMD [95% CI]
Age, mean (SD)	23.5 (2.07)	23.1 (1.84)	0.19	0.17 [0.01–0.41]
Gestational week at pregnancy notification, mean (SD)	14.9 (8.31)	13.8 (7.50)	0.26	0.14 [0.01–0.40]
Educational attainment, n (%)			0.26	0.21 [0.05–0.48]
High school or lower	36 (29.0)	30 (22.9)		
Junior or technical college	42 (33.9)	57 (43.5)		
Bachelor's degree or higher	46 (37.1)	44 (33.6)		
Employment status, n (%)			0.40	0.12 [0.01–0.40]
Unemployed or working ≤ 3 days/week	42 (33.9)	52 (39.7)		
Working ≥ 4 days/week	82 (66.1)	79 (60.3)		
Income, JPY, n (%)			0.47	0.20 [0.09–0.51]
< 4,000,000	40 (32.3)	45 (34.4)		
4,000,000–6,999,999	39 (31.5)	50 (38.2)		
≥ 7,000,000	42 (33.9)	33 (25.2)		
Missing	3 (2.4)	3 (2.3)		
Marital status, n (%)			0.28	0.15 [0.01–0.40]
Married	92 (74.2)	88 (67.2)		
Not married	32 (25.8)	43 (32.8)		
Living arrangements, n (%)			1.00	0.02 [0.01–0.28]
Living with someone	117 (94.4)	123 (93.9)		
Living alone	7 (5.6)	8 (6.1)		
Returned to the maternal home for childbirth, n (%)			0.61	0.13 [0.01–0.31]
No	80 (64.5)	85 (64.9)		
Yes	44 (35.5)	45 (34.4)		
Missing	0 (0.0)	1 (0.8)		
Social support, mean (SD)	4.46 (2.45)	4.13 (2.26)	0.26	0.14 [0.01–0.38]
Missing	2 (1.6)	1 (0.8)		
Perceived fulfillment of psychosocial needs, mean (SD)	28.7 (9.3)	27.1 (10.6)	0.21	0.16 [0.01–0.42]
Well-being, mean (SD)	60.7 (21.7)	62.1 (20.8)	0.59	0.07 [0.00–0.32]

SD, standard deviation; JPY, Japanese yen.

Supplementary Table 4 Changes in perceived fulfillment of psychosocial needs.

	T1<br>< 35 weeks' gestation<br>Difference in mean changes [95% CI]	T2<br>4 weeks after baseline<br>Difference in mean changes [95% CI]	T3<br>1 month postnatal<br>Difference in mean changes [95% CI]	T4<br>6 months postnatal<br>Difference in mean changes [95% CI]	T5<br>12 months postnatal<br>Difference in mean changes [95% CI]
Perceived fulfillment of psychosocial needs					
Early Partnership group changes	0 (Reference)	3.22<br>[1.40 to 5.04]	3.22<br>[1.30 to 5.14]	5.97<br>[4.07 to 7.88]	4.38<br>[2.21 to 6.56]
Usual care group changes	0 (Reference)	0.59<br>[-1.30 to 2.47]	1.30<br>[-0.57 to 3.16]	1.81<br>[-0.15 to 3.78]	0.46<br>[-1.65 to 2.56]
Between-group difference	0 (Reference)	2.63<br>[-0.03 to 5.30]	1.93<br>[-0.82 to 4.67]	4.16<br>[1.35 to 6.97]	3.93<br>[0.63 to 7.23]

CI, confidence interval; NA, not applicable
Missing cases were 0%, 20.5%, 25.2%, 35.8%, and 43.7% at T1–T5 in the Early Partnership group, and 0%, 22.2%, 27.8%, 32.3%, and 37.3% in the Usual Care group. Missing data were handled using multiple imputation; thus, all participants were included in the analyses.

Supplementary Table 5 Between-group difference in postnatal depressive symptoms. Negative values indicate lower levels of postnatal depressive symptoms in the Early Partnership group compared with usual care.

	T3<br>1 month postnatal<br>Mean difference [95% CI]	T4<br>6 months postnatal<br>Mean difference [95% CI]	T5<br>12 months postnatal<br>Mean difference [95% CI]
Postnatal depressive symptoms	-0.57<br>[-1.69 to 0.55]	-1.54<br>[-2.70 to -0.38]	-1.55<br>[-2.74 to -0.36]

CI, confidence interval
Missing cases were 23.8%, 36.4%, and 46.4% at T3, T4, and T5 in the Early Partnership group, and 28.5%, 31.6%, and 38.6% in the Usual Care group. Missing data were handled using multiple imputation; thus, all participants were included in the analyses.

Supplementary Table 6 Changes in perceived fulfillment of well-being.

	T1<br>< 35 weeks' gestation<br>Difference in mean changes [95% CI]	T2<br>4 weeks after baseline<br>Difference in mean changes [95% CI]	T3<br>1 month postnatal<br>Difference in mean changes [95% CI]	T4<br>6 months postnatal<br>Difference in mean changes [95% CI]	T5<br>12 months postnatal<br>Difference in mean changes [95% CI]
Well-being					
Early Partnership group changes	0 (Reference)	7.99<br>[4.18 to 11.80]	10.17<br>[6.44 to 13.89]	13.85<br>[9.95 to 17.75]	15.71<br>[11.40 to 20.01]
Usual care group changes	0 (Reference)	2.93<br>[-0.66 to 6.53]	6.21<br>[2.37 to 10.04]	9.32<br>[5.76 to 12.87]	11.52<br>[7.82 to 15.21]
Between-group difference	0 (Reference)	5.05<br>[-0.26 to 10.37]	3.96<br>[-1.33 to 9.25]	4.53<br>[-0.85 to 9.92]	4.19<br>[-1.60 to 9.98]

CI, confidence interval; NA, not applicable
Missing cases were 0%, 20.5%, 23.8%, 35.8%, and 43.7% in the Early Partnership group, and 0%, 22.2%, 27.8%, 32.3%, and 38.0% in the Usual Care group. Missing data were handled using multiple imputation; thus, all participants were included in the analyses.

Supplementary Table 7 Sensitivity analysis with missing data handled using full information maximum likelihood instead of multiple imputation: Changes in perceived fulfillment of psychosocial needs.

	T1<br>< 35 weeks' gestation<br>Difference in mean changes [95% CI]	T2<br>4 weeks after baseline<br>Difference in mean changes [95% CI]	T3<br>1 month postnatal<br>Difference in mean changes [95% CI]	T4<br>6 months postnatal<br>Difference in mean changes [95% CI]	T5<br>12 months postnatal<br>Difference in mean changes [95% CI]
Perceived fulfillment of psychosocial needs					
Early Partnership group changes	0 (Reference)	3.79<br>[2.00 to 5.58]	3.27<br>[1.44 to 5.09]	6.75<br>[4.83 to 8.67]	5.00<br>[3.01 to 7.00]
Usual care group changes	0 (Reference)	0.39<br>[-1.35 to 2.13]	0.98<br>[-0.82 to 2.77]	1.33<br>[-0.50 to 3.16]	-0.39<br>[-2.27 to 1.49]
Between-group difference	0 (Reference)	3.40<br>[0.91 to 5.90]	2.29<br>[-0.26 to 4.84]	5.42<br>[2.77 to 8.07]	5.39<br>[2.66 to 8.13]

CI, confidence interval; NA, not applicable
Missing cases were 0%, 20.5%, 25.2%, 35.8%, and 43.7% at T1–T5 in the Early Partnership group, and 0%, 22.2%, 27.8%, 32.3%, and 37.3% in the Usual Care group.

Supplementary Table 8 Sensitivity analysis with missing data handled using full information maximum likelihood instead of multiple imputation: Between-group difference in postnatal depressive symptoms. Negative values indicate lower levels of postnatal depressive symptoms in the Early Partnership group compared with usual care.

	T3<br>1 month postnatal<br>Mean difference [95% CI]	T4<br>6 months postnatal<br>Mean difference [95% CI]	T5<br>12 months postnatal<br>Mean difference [95% CI]
Postnatal depressive symptoms	-0.66<br>[-1.89 to 0.58]	-1.99<br>[-3.28 to -0.71]	-1.92<br>[-3.26 to -0.59]

CI, confidence interval
Missing cases were 23.8%, 36.4%, and 46.4% at T3, T4, and T5 in the Early Partnership group, and 28.5%, 31.6%, and 38.6% in the Usual Care group.

Supplementary Table 9 Sensitivity analysis with missing data handled using full information maximum likelihood instead of multiple imputation: Changes in perceived fulfillment of well-being.

	T1<br>< 35 weeks' gestation<br>Difference in mean changes [95% CI]	T2<br>4 weeks after baseline<br>Difference in mean changes [95% CI]	T3<br>1 month postnatal<br>Difference in mean changes [95% CI]	T4<br>6 months postnatal<br>Difference in mean changes [95% CI]	T5<br>12 months postnatal<br>Difference in mean changes [95% CI]
Well-being					
Early Partnership group changes	0 (Reference)	8.79<br>[5.23 to 12.35]	10.50<br>[6.89 to 14.11]	15.60<br>[11.80 to 19.40]	16.70<br>[12.80 to 20.70]
Usual care group changes	0 (Reference)	3.06<br>[-0.41 to 6.52]	6.71<br>[3.15 to 10.27]	10.20<br>[6.52 to 13.88]	12.80<br>[9.07 to 16.53]
Between-group difference	0 (Reference)	5.73<br>[0.76 to 10.70]	3.79<br>[-1.27 to 8.86]	5.45<br>[0.19 to 10.70]	3.95<br>[-1.48 to 9.38]

CI, confidence interval; NA, not applicable
Missing cases were 0%, 20.5%, 23.8%, 35.8%, and 43.7% in the Early Partnership group, and 0%, 22.2%, 27.8%, 32.3%, and 38.0% in the Usual Care group.
